# Single primer isothermal amplification (SPIA) combined with next generation sequencing provides complete bovine coronavirus genome coverage and higher sequence depth compared to sequence-independent single primer amplification (SISPA)

**DOI:** 10.1371/journal.pone.0187780

**Published:** 2017-11-07

**Authors:** Mette Myrmel, Veslemøy Oma, Mamata Khatri, Hanne H. Hansen, Maria Stokstad, Mikael Berg, Anne-Lie Blomström

**Affiliations:** 1 Department for Food Safety and Infection Biology, Norwegian University of Life Sciences, Oslo, Norway; 2 Department of Production Animal Clinical Sciences, Norwegian University of Life Sciences, Oslo, Norway; 3 CIGENE, Norwegian University of Life Sciences, Ås, Norway; 4 Department of Biomedical Sciences and Veterinary Public Health, Section of Virology, Swedish University of Agricultural Sciences, Uppsala, Sweden; Oklahoma State University, UNITED STATES

## Abstract

Coronaviruses are of major importance for both animal and human health. With the emergence of novel coronaviruses such as SARS and MERS, the need for fast genome characterisation is ever so important. Further, in order to understand the influence of quasispecies of these viruses in relation to biology, techniques for deep-sequence and full-length viral genome analysis are needed. In the present study, we compared the efficiency of two sequence-independent approaches [sequence-independent single primer amplification (SISPA) and single primer isothermal amplification (SPIA, represented by the Ovation kit)] coupled with high-throughput sequencing to generate the full-length genome of bovine coronavirus (BCoV) from a nasal swab. Both methods achieved high genome coverage (100% for SPIA and 99% for SISPA), however, there was a clear difference in the percentage of reads that mapped to BCoV. While approximately 45% of the Ovation reads mapped to BCoV (sequence depth of 169–284 944), only 0.07% of the SISPA reads (sequence depth of 0–249) mapped to the reference genome. Although BCoV was the focus of the study we also identified a bovine rhinitis B virus (BRBV) in the data sets. The trend for this virus was similar to that observed for BCoV regarding Ovation vs. SISPA, but with fewer sequences mapping to BRBV due to a lower amount of this virus. In summary, the SPIA approach used in this study produced coverage of the entire BCoV (high copy number) and BRBV (low copy number) and a high sequence/genome depth compared to SISPA. Although this is a limited study, the results indicate that the Ovation method could be a preferred approach for full genome sequencing if a low copy number of viral RNA is expected and if high sequence depth is desired.

## Introduction

Next generation sequencing (NGS) has become a valuable tool in virology studies and has the power to generate whole genome sequences (WGS) from small amounts of virus, rapidly and at a relatively low cost [1]. Although direct WGS of the foot-and-mouth disease virus (FMDV) has been reported [2], viral RNA amounts in clinical samples are usually too low for direct WGS. Amplification of the nucleic acid is usually required and sequence-dependent and independent methods are available. A common method for amplifying viral RNA is RT-PCR using virus specific primers. This method is, however, cumbersome due to the requirement of multiple sets of primers to cover the full viral genome. Sequence-independent methods are attractive in that they allow sequencing of highly divergent viruses, but efficient reduction of background nucleic acids is required in order to get good coverage and depth (number of times each nucleotide is sequenced) of the viral genomes [3]. Using standard Sanger sequencing only the dominating strain(s) is(are) sequenced. In contrast, NGS yields deep sequencing meaning that each nucleotide in the sample is sequenced several times. While standard sequencing misses allelic variants with a frequency below 20% in a population [4], NGS can detect less abundant variants depending on sequencing depth. This provides an increased opportunity for viral metagenomics (study of the total viral community), for epidemiologic surveillance of viruses, and for obtaining insight into viral evolution and fitness. Infectious agents, and especially RNA viruses, can rapidly change and adapt to their host and produce many variants (quasispecies) [1, 5–8]. In order to identify these variants, thousands of sequences are needed, which makes NGS particularly useful. An RNA virus family that has gained specific interest, through the emergence of SARS and MERS as well as through containing a number of animal pathogens, is *Coronaviridae* [9]. Coronavirus have high mutation rates and are prone to recombination [10]. Virus quasispecies composition with negative and positive interactions among mutants, is the source of virus evolution during “group selection” and influences the biological behaviour of a viral population [5, 11]. Therefore, it is important not only to have techniques that can rapidly sequence new emerging coronaviruses but also those that investigate the quasispecies population in order to understand the biology of these important viruses.

The aim of the present study was to evaluate two different sequence-independent methods for coronavirus RNA/cDNA amplification. Sequence-independent single primer amplification (SISPA) and NuGEN’s Ovation RNA-seq system v2 (single primer isothermal amplification-SPIA) were tested in order to obtain complete genomes of a bovine coronavirus (BCoV) from a nasal swab using Illumina for sequencing. Focus was put on total reads, total BCoV reads, read depth and genome coverage.

## Materials and methods

### Sampling, extraction of RNA and RT-qPCR

A nostril specimen was collected from a calf naturally exposed to BCoV during a transmission study [12]. Briefly, a flocked eSwab^™^ (Copan Diagnostics, CA, USA) was swept inside the calf’s nostril and kept frozen in 1 ml transport medium at -80°C until analysis. The specimen was thawed on ice and the swab medium centrifuged at 9700 x g for 10 min. Two technical replicates were processed individually until library preparation and NGS. The supernatant (140 μl) was treated with 2.8 μg RNase A (Sigma-Aldrich, MO, USA) and 6 Units Turbo DNase (Ambion, MA, USA) for 30 min at 37°C. Thereafter RNA was extracted with QIAzol (Qiagen, Hilden, Germany) and chloroform phase separation. The RNA containing aqueous phase was mixed with 70% ethanol (1:1) and added to an RNeasy Mini Kit column (Qiagen), according to the manufacturer. The RNA concentration (3–5 ng/μl) and purity were measured by Nanodrop (Thermo Scientific, DE, USA) and the RNA was kept at -80°C until further use. Estimation of the BCoV copy number (4,1 x 10^5^ genome copies used) was done with RT-qPCR using primers and probe as described by Decaro and colleagues [13]. A tenfold dilution series of a plasmid containing the BCoV amplicon was used as a quantification standard. Quantification of a bovine rhinitis virus (700 genome copies used) that was incidentally found in the nasal sample, was performed in an identical way after establishing a rhinitis virus RT-qPCR and production of a plasmid containing the rhinitis virus target.

### SISPA—Sequence independent single primer amplification (sample S1 and S2)

A variant of the SISPA protocol was followed [14]. Ten μl RNA was reverse transcribed and tagged using 10 μM primer FRoV26-N (GCC GGA GCT CTG CAG ATA TCN NNN NN) [15] and Superscript III (Invitrogen, MA, USA) according to manufacturer’s instructions. Second strand was made by adding 0.5 μl of Klenow fragment (3’ -> 5’ exo-) (New England Biolabs, England) to the cDNA and incubation at 37°C for 60 min and 75°C for 10 min. Double stranded (ds) DNA was kept at -20°C prior to PCR amplification.

Three aliquots of 6 μl tagged dsDNA were amplified using 0.8 μM of primer FR20 (GCC GGA GCT CTG CAG ATA TC), 1 U KOD DNA polymerase (Merck Millipore, Darmstadt, Germany), 1 mM MgCl_2_ and 0.2 mM dNTP. The amplification steps were 95°C for 20 s followed by 30 cycles at 95°C for 30 s, 58°C for 30 s, 72°C for 90 s and completed at 72°C for 10 min. The PCR products were purified with the NucleoSpin^®^ Gel and PCR clean-up kit (Macherey-Nagel, Düren, Germany), according to the manufacturer’s protocol for products in solution, and eluted in 45 μl buffer. Primer sequences were cleaved off using 30 U EcoRV (Promega, WI, USA) and incubation at 37°C for 60 min, before pooling of aliquots and purification using NucleoSpin^®^ Gel and PCR clean up kit with a final elution volume of 65 μl. The DNA concentration was measured using Nanodrop (50–52 ng/μl).

### SPIA—Single primer isothermal amplification (Ovation, sample O1 and O2)

Five μl RNA was subjected to cDNA synthesis and amplification using the Ovation RNA-Seq System V2 (NuGen, CA, USA) following the manufacturer’s instructions. In short, cDNA was produced using oligo dT and random hexamers. DsDNA was generated, purified using Agencourt RNAClean XP beads (Beckman Coulter, CA, USA) and amplified on beads using single primer isothermal amplification (SPIA) [16]. The SPIA reaction was 4°C for 1 min, 47°C for 60 min, 80°C for 20 min and hold at 4°C. After removing the beads, 40 μl of amplified dsDNA was purified with Qiaquick PCR purification Kit (Qiagen) and eluted in 30 μl of buffer. The DNA concentration was measured by Nanodrop (104–108 ng/μl).

### Library preparation and sequencing

Four libraries (representing S1-2 and O1-2) were prepared for sequencing using Illumina Nextera XT DNA Library Preparation Kit (Illumina, CA, USA). In order to (i) achieve a high concentration of nucleic acid for sequencing and (ii) remove primer-dimers from the PCR product, slight modifications to the manufacturer’s protocol were made; (i) using 1,5 ng total input DNA and (ii) an extra clean-up cycle after the PCR step, with 1.5 x ratio Agencourt AMPure XP Beads (Beckman Coulter). Libraries were validated by quantification using Qubit^®^ 2.0 Fluorometer and Qubit^®^ dsDNA HS Assay kit (Invitrogen) and size analysis using a 2100 BioAnalyzer system with the DNA High Sensitivity kit (Agilent Technologies, CA, USA). The mean library concentration and length were 1.5 ng/μl and 740 bp, respectively. For normalization, the libraries were diluted to 2 nM using the equation

$$Molarity=\frac{ng/\mu l\;x\;10^{6}}{660\;x\;Average\;library\;fragment\;length}$$

Finally equal amounts of each library were pooled. Sequencing was performed using a MiSeq (Illumina) and 600 Cycles MiSeq Reagent Kit v3 in a paired-end mode.

### Data analysis

Raw data from the MiSeq run were quality checked and trimmed (Q≥30; max number of ambiguities = 2) using CLC Genomic workbench (v7.5.3) (Qiagen) in order to remove poor data. Reads that passed the quality criteria were mapped against a reference BCoV genome available in GenBank (strain Mebus, accession number U00735.2) using the reference mapping tool (default values) in the CLC Genomic workbench. Sequences not mapping to BCoV were annotated through blastx analysis using Diamond [17]. The Diamond was run in sensitive mode and the blastx was performed against the nr-database (NCBI) using an e-value cut-off at 0.0001. SortMeRNA [18] was used to characterise the ribosomal RNA (rRNA) composition of each dataset and was run against the following databases: rfam 5.8s, rfam 5s, silva arc 16s, silva arc 23s, silva bac 16s, silva bac 23s, silva euk 18s and silva euk 28s.

## Results

### Sequence data

The majority of the reads from all four datasets remained after quality trimming although the ends were trimmed making the average read length 175–184 nt (Table 1). Mapping of the reads to the BCoV reference revealed a clear difference between the datasets originating from the two amplification methods, as only 0.07% of the reads from the SISPA datasets mapped to BCoV compared to 42–47% of the reads from the Ovation datasets (Table 1).

**Table 1:** Sequence data output and results of the bovine coronavirus mapping.

Reads	Data sets			
Raw	O1	O2	S1	S2
Total reads	9 231 412	6 353 426	6 930 526	6 271 024
Average length (nt)	216,4	228,6	246,5	256,6
**Trimmed**				
Total reads	9 190 660	6 325 015	6 907 172	6 258 362
Average length (nt)	175,0	183,6	175,8	182,2
**Mapped to BCoV**				
Total reads	3 891 314	3 027 739	4 554	4 654
% mapped reads	42,34	47,87	0,07	0,07
Range of depth	254–288 944	164–202 537	0–208	0–249

One nasal swab from an infected calf was processed in duplicate with the Ovation (O) and the SISPA (S) protocols.

Regarding the non-BCoV sequences, for both methods the majority (87–89%) could be annotated through blastx as eukaryotic and 10–13% were of bacterial origin. SortMeRNA classified approximately 11% of all the reads from each of the Ovation datasets as rRNA and of these eukaryotic 18s and 28s were most abundant. In the SISPA datasets, nearly 8% were classified as rRNA with half of the sequences mapping to the rfma 5s database and the other half to the 18s and 28s eukaryotic databases.

Interestingly, apart from a low number of phage and retrovirus sequences, another +ssRNA virus was identified in all samples—a bovine rhinitis B virus (BRBV). In O1 and O2 between 9 000–11 000 reads were identified covering most of the BRBV genome, while in S1 and S2, only 3 and 10 sequences, respectively, were identified. The BRBV identified has been genetically characterised [19]. The difference in overall viral content between the data sets generated with the two amplification methods was obvious and Table 2 shows the combined classification of the reads annotated through BCoV mapping and blastx.

**Table 2:** Distribution of annotated sequences. Reads were classified through bovine coronavirus mapping and blastx. The table shows percentage of reads from the ovation (O) and SISPA (S) samples that mapped to bacteria, archaea, eukarya and virus.

	Data sets			
	O1	O2	S1	S2
**Bacteria**	2,45	2,25	11,94	12,93
**Archaea**	0,0005	0,0011	0,0002	0
**Eukarya**	21,81	19,20	88,03	87,03
**Virus**	75,72	78,55	0,02	0,01

### BCoV coverage and sequence depth

**Fig 1:**
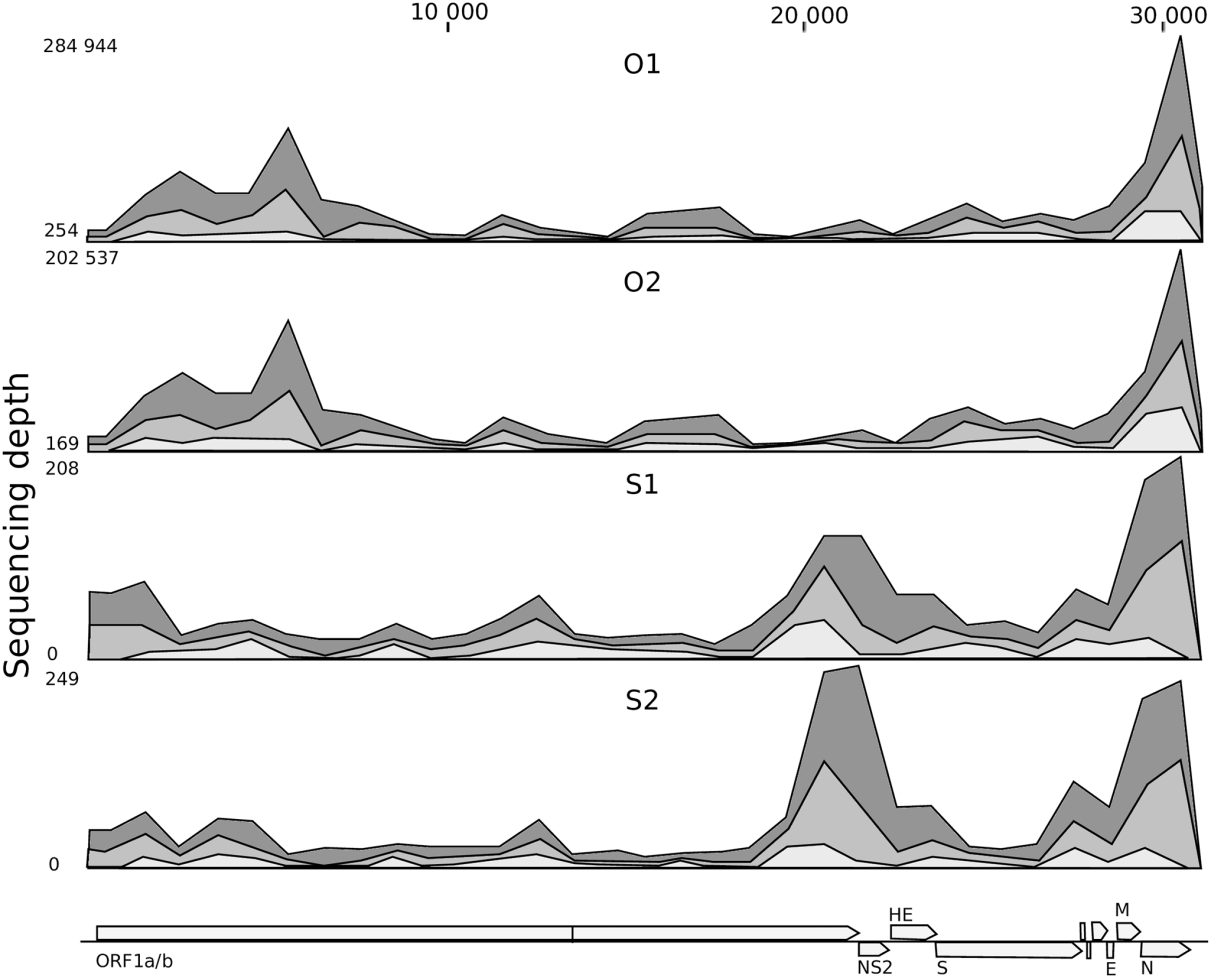
Coverage and sequence depth of the samples; O1 and O2 (ovation); S1 and S2 (SISPA). The lower part shows the annotation of the bovine coronavirus (strain Mebus) used in the mapping. The different shading of grey shows the minimum, mean and maximum depth values over a 1000 bp region.

For the SISPA samples (S1 and S2), coverage of the BCoV genome was 99% and the 5’ and 3’ untranslated regions (UTRs) were not complete. Also, in the S2 assembly a number of short regions towards the middle of the genome lacked coverage. Unlike the SISPA method, BCoV coverage for the Ovation samples (O1 and O2) was complete, including both UTRs (Fig 1). The consensus sequences of BCoV in O1 and O2 were identical.

When comparing data from the two amplification approaches, there was a clear difference between number of reads mapping to the reference BCoV genome and sequence depth, despite the high similarity in genome coverage and the total number of reads (Table 1). For both SISPA sets, only 0.07% of reads mapped to the reference genome and the sequence depth varied between 0–208 for S1 and 0–249 for S2. In contrast, 42.34% (O1) and 47.87% (O2) of the reads from samples prepared by the Ovation protocol mapped to the BCoV genome. Sequencing of the Ovation samples was, therefore, much deeper and displayed a depth of 254–284 944 (S1) and 169–202 537 (S2). Hence, the lowest sequence depth for the Ovation samples was comparable to the highest depth for SISPA. All data sets displayed a peak at the 3´end of the genome (N-gene) (Fig 1). For the Ovation samples a peak showed at 6 kb (ORF1a), while in the SISPA samples there was a peak just after 20 000 bp (end of ORF1/b and covering the NS2 gene). Also towards the 5´end of all samples, an increased sequence depth was found compared to the middle part of the genome.

## Discussion

High-throughput sequencing has provided the possibility of sequencing not only the consensus sequence of a virus but the viral cloud (quasispecies) that exists in a particular sample [7]. For RNA viruses this is of extra importance as these viruses have a high mutation rate and exist, in an individual, as a population of related viruses which may affect the viral fitness, host specificity and pathogenesis [5, 11]. Coronaviruses are the largest RNA viruses known (27–32 kb positive sense RNA) and are considered emerging pathogens in both humans and animals [9]. In order to understand the biology of coronaviruses, it is important to have tools that can generate full-length genome sequences of emerging coronaviruses as well as provide information on the quasispecies population. Direct WGS of the FMDV has been performed on samples containing ≥ 1 x 10^7^ genome copies per ul RNA [2]. However, as BCoV has a larger RNA genome (32 kb compared to 8.5 kb for FMDV) and our clinical samples contained less viral RNA, pre-amplification of the RNA was considered a necessity.

The present study compared two methods, SISPA and Oviation, for sequence-independent amplification of BCoV RNA. SISPA was chosen as it is a commonly used method for virus detection and has also been used for full-length viral genome sequencing [20]. The method includes tagged random primers to produce/label cDNA/DNA prior to PCR targeting the tag-sequence [15]. Ovation, on the other hand, is based on single-primer isothermal linear amplification (Ribo-SPIA) [21] and has been shown to generate full-length genomes of HIV, respiratory syncytial and West Nile virus from as little as 100 viral RNA copies [22].

In the present study, the two amplification methods gave approximately the same total number of reads and a very high coverage of the BCoV genome. While Ovation gave complete coverage, the SISPA approach missed parts of the UTRs as well as short regions in the middle of the genome. Although both methods produced sequences that covered all or most of the BCoV genome, there was a clear difference in the percentage of reads mapping to the reference genome. While less than 1% of the reads from the SISPA data sets mapped to BCoV, around 45% of the Ovation reads did. As most of the total SISPA reads (˃ 99%) originated from eukaryotes and bacteria and only 8% were classified as rRNA, filtration of the sample as well as an additional DNase treatment of the RNA could have resulted in more viral reads using the SISPA protocol. However, the much higher ratio of BCoV reads (approx. 45%) from the Ovation data sets compared to 0,07% still indicates a significant advantage of this amplification method compared to the SISPA protocol. The benefit of the Ovation method is not restricted to deep sequencing of BCoV as this protocol also gave roughly 1000 times more BRBV reads than the SISPA protocol.

The large difference in number of reads that mapped the BCoV reference genome resulted in a significant difference in sequence depth between the methods. The SISPA dataset had a maximum depth of 249 compared to 284 944 for the Ovation method. This difference was also found for BRBV as Ovation gave a sequence depth of 0–1000 covering the majority of the genome, while 3 and 10 sequences in total were present in the two SISPA data sets. The lower sequencing depth of BRBV was probably due to the lower number of BRBV genomes in the sample (1 to 600 compared to BCoV genomes).

The pattern of sequence depth was almost identical for the technical replicates O1 and O2 and was very similar for replicates S1 and S2 (Fig 1). An uneven depth was seen across the genome for both methods with a sequence depth peaking at the 3’ end of the genome. For quasispecies analysis, a difference in sequence depth across the genome could pose a problem, as the possibility to compare variation in different regions will be reduced. The Ovation kit includes poly-T primers in addition to random (6N) primers, this could cause the 3’end peak, but does not explain the peak in the SISPA dataset. Fragmentation is a factor that may influence the variation of sequence coverage. However, in a study by Knierim et al. (2011) three different fragmentation methods (nebulization, sonication and enzymatic) gave similar coverage patterns [23]. Rosseel et al. (2013) got similar results, as they did not observe any differences when comparing fragmented to unfragmented samples. According to Malboeuf et al. (2012) depth variability across a genome could be due to secondary RNA structures [22], but this was not confirmed by Rosseel et al. who found biased annealing of the random primer, caused by the tag sequence, to influence SISPA results the most [24]. Extending the random sequence (from 6N to 12 N) and including more than one primer tag, may reduce the bias, for both methods.

Although the present study is limited, the results on two technical replicates show a clear difference between the two methods regarding efficient amplification of the RNA genome from two different viruses. Also, as amplification of nucleic acids is prone to introduction of errors in the nucleotide sequence, analysis of two technical replicates enabled some control of the sequencing quality. Focus was put on the consensus sequences, as these are anticipated to be identical in technical replicates. This was also the result for O1 and O2 replicates and indicates that Ovation is a feasible method for reconstructing the genome haplotype of a 32 kd BCoV.

In summary, the high amounts of BCoV and BRBV reads using the Ovation system indicate a high efficiency of this method for amplification of viral RNA from high and low copy number samples, compared to the SISPA protocol.

## Disclaimer

None of the authors are connected to Nugen. The material contained in this study has not been sponsored by Nugen.
